# Behind the Wheel of Epithelial Plasticity in KRAS-Driven Cancers

**DOI:** 10.3389/fonc.2019.01049

**Published:** 2019-10-11

**Authors:** Emily N. Arner, Wenting Du, Rolf A. Brekken

**Affiliations:** ^1^Cancer Biology Graduate Program, Department of Surgery and the Hamon Center for Therapeutic Oncology Research, University of Texas Southwestern Medical Center, Dallas, TX, United States; ^2^Department of Pharmacology, University of Texas Southwestern Medical Center, Dallas, TX, United States

**Keywords:** EMT, KRAS, metastasis, TBK1, AXL, drug resistance

## Abstract

Cellular plasticity, a feature associated with epithelial-to-mesenchymal transition (EMT), contributes to tumor cell survival, migration, invasion, and therapy resistance. Phenotypic plasticity of the epithelium is a critical feature in multiple phases of human cancer in an oncogene- and tissue-specific context. Many factors can drive epithelial plasticity, including activating mutations in *KRAS*, which are found in an estimated 30% of all cancers. In this review, we will introduce cellular plasticity and its effect on cancer progression and therapy resistance and then summarize the drivers of EMT with an emphasis on KRAS effector signaling. Lastly, we will discuss the contribution of cellular plasticity to metastasis and its potential clinical implications. Understanding oncogenic KRAS cellular reprogramming has the potential to reveal novel strategies to control metastasis in KRAS-driven cancers.

## Introduction

*KRAS* is mutated in an estimated 30% of all cancers. In fact, the small GTPase KRAS has an activating point mutation in over 90% of pancreatic cancer patients (1), ~35% of lung cancer patients, and ~40% of colorectal cancer patients (2). As such, oncogenic KRAS is established as a driver of cancer initiation, progression, metastasis, therapy resistance, and immune suppression in multiple cancers (3). KRAS is an alluring therapeutic target, yet strategies targeting KRAS have been largely unsuccessful. However, understanding downstream effectors of KRAS signaling might provide alternative strategies to indirectly target KRAS and the cellular reprogramming driven by oncogenic KRAS signaling.

Recent evidence suggests that individual KRAS mutations activate distinct signaling pathways (2, 4). For example, gene expression analysis of primary human NSCLCs expressing G12C or G12V activating mutations in KRAS showed distinct gene expression profiles compared to cell lines expressing other KRAS activating point mutations (5). Similarly, Hammond et al. (6) engineered SW48 colorectal cancer cells, which are KRAS wild-type, to express KRAS point mutations: G12V, G12D, or G13D. Subsequent phosphoprotein expression analysis revealed the activation of differential signaling pathways in distinct KRAS mutational contexts. In support of these results, a large-scale screening effort using RNAi, small-molecules, and genetic analysis of cell lines and TCGA analysis revealed that KRAS binds to different effector proteins depending on the cellular context, which was determined by cell lineage, secondary mutations, and metabolic state (7). To further study context-dependent KRAS signaling in cancer, Brubaker et al. (4) developed a statistical approach to humanize multiplexed quantitative proteomic data from mouse models of colon and pancreatic cancer. Through the integration of proteomics and mutation data from human PDAC cohorts they identified synthetic lethal partners with oncogenic KRAS and mutant KRAS tissue-specific and cross-tissue signaling. Each of these studies indicate that the signaling outcome and thus cellular phenotype driven by KRAS mutation is deeply dependent on cellular context.

Epithelial plasticity or an epithelial-to-mesenchymal transition (EMT) is a key cellular program that can be activated by KRAS. EMT contributes to tumor progression by enhancing tumor cell survival and therapy resistance and by facilitating success in the metastatic cascade. In this review, we will introduce cellular plasticity and its effect on cancer progression and therapy resistance and then summarize drivers of EMT with an emphasis on KRAS signaling. Lastly, we will discuss the contribution of cellular plasticity to metastasis and its potential clinical implications.

## Cellular Plasticity and EMT

Cellular plasticity serves as a mechanism of tissue adaptation and regeneration in normal tissues and can also predispose tissue to cancer transformation (8). In the pancreas, pancreatic epithelial and acinar cells display robust plasticity, enabling adaptation to metabolic and environmental stress. In pancreatic cancer, tumor cells alter their phenotype as a result of exposure to diverse metabolic conditions, signaling molecules, stromal elements, and therapeutic agents. This plastic state in tumor cells can facilitate tumor progression, including metastasis, chemoresistance, and immune evasion (8).

Acinar-to-ductal metaplasia (ADM) (9), describes a process where normal pancreatic acinar cells assume a duct-like state in the setting of chronic injury, such as pancreatitis. When pancreatitis resolves in normal/non-malignant pancreatic tissue, ADM lesions revert to acinar morphology. However, if KRAS-transformed acinar cells are subjected to the stress of pancreatitis, precancerous pancreatic intraepithelial neoplasia often forms (10–14). This suggests that pancreatic ductal adenocarcinomas (PDACs) may arise from acinar cells that have undergone transdifferentiation to a duct-like state. Normal pancreatic cells are sensitive to the transforming effects of mutant *KRAS* and the loss of phosphatase and tensin homolog (15), indicating that the likelihood of tumor formation and eventual histologic tumor type depends on the specific drivers that are present as well as the cellular compartments in which they are expressed (16–20).

EMT is another example of cellular plasticity program that is used by cells and tissues to adapt to cues or cellular stress. EMT classically defined is a developmental program that is instrumental in early embryo patterning during gastrulation (21, 22) and is characterized by epithelial cells losing cell-to-cell adhesion, epithelial tight junctions, and desmosomes. These changes are thought to occur through coordinated genetic reprogramming induced by EMT-transcription factors (EMT-TFs) that are activated in response to extracellular cues (21). These cues include growth factors such as transforming growth factor-β (TGFβ), epidermal growth factor (EGF), hepatocyte growth factor (HGF), and insulin-like growth factor 1 (IGF1) (21, 23–26). This essential developmental program can be hijacked during tumorigenesis to promote increased cell migration and survival.

**Figure 1:**
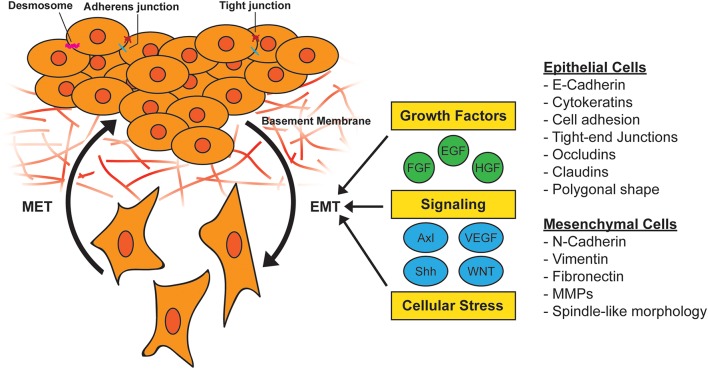
Activation of epithelial-to-mesenchymal transition (EMT). During EMT, epithelial cells lose their cell-to-cell adhesion and adopt a more spindle-like morphology due to the expression of mesenchymal markers. This morphology change results in the ability to escape the basement membrane and invade and survive stressful situations, including therapy. EMT can be induced by a variety of growth factors, signaling pathways, and cellular stress such as hypoxia and nutrient deprivation. MET, mesenchymal-to-epithelial transition; MMP, matrix metallopeptidase.

EMT in tumor cells can also be induced by cellular stress such as inflammation or nutrient/oxygen deprivation (27), and transforming oncogenes including oncogenic *KRAS* (28, 29). The genetic reprogramming associated with EMT in normal tissue or cancer leads to a shift from an epithelial to a mesenchymal phenotype. Epithelial cells often have polygonal shapes in monolayer culture, are polarized along their apical-basal axis and are tightly joined to one another laterally through adherens junctions. In contrast, mesenchymal cells exhibit spindle-like morphology and are loosely attached to the surrounding stroma through focal adhesions, which contributes to increased motility and invasive behavior (30) (Figure 1).

In epithelial tumors, the manifestation of an EMT program is associated with tumor grade. High-grade cancer is aggressive and characterized by a loss of normal tissue structure and architecture. High-grade tumors are often described as poorly differentiated and mesenchymal, displaying tumor cells that have undergone EMT. In contrast, low-grade tumors are characterized as well-differentiated cancers that retain an epithelial phenotype. Across human cancer, tumors that are high grade and poorly differentiated carry a worse prognosis with a high likelihood of metastasizing to distant organs (8).

EMT is a common feature associated with tumor progression and is thought to be critical to cancer cell dissemination in some tumors (31–33). The metastasis of epithelial tumors, such as PDAC, requires the cancer cells to escape epithelial nests, invade surrounding stroma, intravasate into blood or lymphatic vessels, survive circulation, and extravasate at the secondary site, where successful cells form micrometastases and eventually macrometastases (34). The escape of tumor cells from tumor cell nests encapsulated by a basement membrane can be facilitated by tumor cell epithelial plasticity, which results in epithelial tumor cells losing contact with the basement membrane and nearby cells while adopting mesenchymal-like features that enable cell migration and invasion. This is a common feature in mouse models of PDAC (35–37). While epithelial plasticity alters morphology and cell-cell contact it also enhances tumor cell survival under stressful environmental conditions, such as chemotherapy and radiation (32, 38–40). EMT and metastasis are generally considered to be late events in tumorigenesis; however, EMT and the metastatic cascade has been shown to occur even in “preinvasive” stages of PDAC (35). Thus, the concept that EMT is driven by the oncogenotype of a tumor is worthy of consideration.

In KRAS-driven tumors, such as PDAC, tumorigenesis and epithelial plasticity programs are often intertwined. For example, in genetically engineered mouse models (GEMMs) of PDAC harboring mutant KRAS, EMT was found to be an early event after tumor formation (35). Furthermore, co-expression of mutant KRAS and a polycomb-group repressor complex protein, Bmi1, in normal human pancreatic duct-derived cells (HPNE) induces partial EMT via upregulation of the EMT-TF Snail (28, 41–43). In addition, multiple receptor tyrosine kinases (RTKs) implicated in the induction of EMT activate RAS and the resulting signaling cascade induces the expression of EMT-TFs in a RAS-dependent manner (43–46). Other pathways have also been shown to interact with mutant KRAS to drive EMT. For example, the EMT-TF, Snail has been shown to induce TGFβ signaling in a mutant KRAS dependent manner to drive EMT (47). Other studies revealed that signal transducer and activator of transcription 3 (STAT3) can mediate a synergistic interaction between TGFβ and RAS resulting to enhance Snail driven EMT (48). Other small GTPases, RAC, and RHO, are also activated by RAS via PI3K to drive EMT by regulating adherens junctions and focal adhesions (49). Thus, while mutant KRAS driven tumors are often dependent on RAS activity for development and maintenance (28, 41, 42) the prominent oncogenic mutation also is a critical component of epithelial plasticity.

## EMT and Therapy Resistance

Epithelial plasticity is a key chemoresistance and immune surveillance evasion strategy exploited by tumor cells (50, 51). Plastic tumor cells exhibit increased rates of resistance to therapy including radio-, chemo-, targeted, and immunotherapy (39, 40, 52–54). Stress, such as inflammation, nutrient/oxygen deprivation, and therapy can induce epithelial plasticity in cancer cells (27). A common consequence of EMT is reduced drug uptake by tumor cells. For example, the expression of equilibrative nucleoside transporter 1 (ENT1), which can transport nucleoside analog chemotherapy into cells, is often reduced in tumor cells that have undergone EMT. However, tumors engineered to lack EMT transcription factors (EMT-TFs), such as Snail and Twist, showed elevated ENT1 expression and increased sensitivity to gemcitabine, a nucleoside analog (55). Consistent with these results, Ludwig et al. (54) found that inhibition of AXL reduced epithelial plasticity in models of PDAC, increased ENT1 expression and enhanced sensitivity to gemcitabine when compared to gemcitabine alone or control treated animals. To combat chemoresistance in cancer patients, intermittent dosing or “drug holidays” have been suggested, although recent studies have revealed that resistance driven by oncogenic KRAS is not reversible (56). In human cancer cell lines, therapy resistance driven by mutant *KRAS* was found to irreversibly drive ZEB1-dependent EMT and chemoresistance through the hyperactivation of ERK1/2 (56), arguing against the use of intermittent dosing in tumors driven by oncogenic KRAS. Fischer et al. (57) showed in a spontaneous breast-to-lung metastasis model that EMT contributes to chemotherapy resistance, as mesenchymal-like tumor cells survived cyclophosphamide treatment, demonstrating reduced proliferation, apoptotic tolerance, and increased expression of chemoresistance-related genes. These observations highlight the potential increase in therapeutic efficacy that might result from combining standard therapy with strategies to combat epithelial plasticity.

The hypoxic state of pancreatic tumors increases tumor cell migration and chemoresistance (58). In fact, EMT can be driven by hypoxia often via the induction of TGFβ (59). Additionally, in human pancreatic cancer cell lines, hypoxia has been shown to drive EMT in an NFκB dependent manner through the stability of hypoxia-inducible factor 1 alpha (HIF-1α) and subsequent activation of RelA (p. 65) (60–63), a subunit of the NFκB family of transcription factors (64, 65). NFκB is considered a crucial component of drug resistance in mutant KRAS driven tumors such as pancreatic cancer and colorectal cancer, which typically expresses high levels of the protein (66). The activation of NFκB has been shown to upregulate anti-apoptosis proteins such as Bcl-XL and Bcl-2, promoting chemoresistance (67, 68). As such, NFκB inhibition might be an approach to combat chemoresistance in tumors with KRAS-driven EMT.

Resistance to targeted therapy has also been associated with a mesenchymal state. In non-small cell lung cancer (NSCLC), the expression of an EMT gene signature, which included AXL expression, was associated with resistance to treatment with epidermal growth factor receptor (EGFR) and phosphatidylinositol 3-kinase (PI3K) inhibitors (69–73). Similarly, *in vitro* studies suggested that epithelial NSCLC cell lines are more sensitive to EGFR inhibitors than mesenchymal cell lines (74), and that when AXL is inhibited, sensitivity to EGFR inhibitors is increased (75, 76). In breast cancer patients, the EMT program also serves as a major driver of drug resistance, disease occurrence, and systemic dissemination (52, 77, 78).

In addition to targeted and chemotherapy, EMT has been associated with resistance to immunotherapy (79). In murine melanoma cells, Snail, a canonical EMT-TF, was found to be necessary and sufficient for resistance to cytotoxic T-cell–mediated killing via the induction of regulatory T cells. The effect was driven by immunosuppressive CD11c^+^ dendritic cells, which were generated in response to Snail-expressing melanoma cells (40). Similarly, immune therapy-resistant melanomas display a mesenchymal gene signature, including the downregulation of E-cadherin and upregulation of factors involved in extracellular matrix (ECM) remodeling, angiogenesis, and wound healing (80). Additionally, the immune system is a key component of chemotherapy responses, as many chemotherapeutic agents directly affect the immune landscape of tumors (81). Therefore, identification of key signaling pathways involved in epithelial plasticity could reveal overlap with tumor immune evasion and new therapeutic targets, inhibition of which increases the efficacy of chemo- and immunotherapy.

## EMT and Tumor Metabolism

Metabolic alterations are associated with mutant KRAS-induced EMT. Cancer cells often increase glycolytic flux to meet the high energy demand to support rapid cell growth and division (82). In contrast to normal cells that typically generate energy via the breakdown of pyruvate, cancer cells generate energy by the non-oxidative breakdown of glucose with tumor cells displaying glycolytic rates up to 200 times higher than normal cells in the body (83). This preferential activation of glycolysis for energy supply is referred to as the “Warburg Effect” (83). In pre-clinical models as well as human patient samples, oncogenic Kras signaling can transcriptionally upregulate the glucose transporter GLUT1, as well as multiple enzymes in the glycolytic pathway [e.g., Hexokinase1 (HK1), Hexokinase2 (HK2), Phosphofructokinase1 (PFK-1), and Lactate dehydrogenase A (LDHA)] (82, 84, 85). Hypoxia, a common environmental condition in solid tumors, triggers O-linked β-N-acetylglucosamine (O-GlcNAcylation) at S529 of PFK-1, inducing glycolysis and giving a selective growth advantage to the cancer cells (86, 87). Cancer induced HIF-1α and MUC1 have also been shown to upregulate the expression of key glucose transporters and glycolytic enzymes, including GLUT1 and aldolase A, which leads to increased glucose uptake and glycolysis (82, 84, 88). In addition to glycolysis, recent evidence suggests oncogenic KRAS drives glucose into the hexosamine biosynthetic pathway (HBP), which is required for multiple glycosylation events (89, 90). Taparra et al. (91), recently showed in models of lung tumorigenesis, that KRAS and the EMT program coordinated elevated expression of key enzymes within the HBP pathway. Additionally, they showed that elevated O-GlcNAcylation of intracellular proteins such as the EMT-TF Snail results in suppressed oncogenic-induced senescence and accelerated lung tumorigenesis (91). Understanding the evident metabolic changes driven by oncogenic KRAS and reinforced by epithelial plasticity may reveal novel therapeutic targets for KRAS-driven tumorigenesis.

## Drivers of EMT

A variety of stimuli can induce EMT, including soluble factors, ECM components, environmental conditions, and oncogenic transcriptional programs (92). These stimuli, which include signaling factors such as TGFβ, Wnt, Notch, and Sonic hedgehog (Shh), as well as growth factors such as EGF and platelet-derived growth factor (PDGF) and vascular endothelial growth factor (VEGF), serve as ligands for the signaling pathways they activate (Figure 1). EMT programs can also be activated in response to several paracrine signals in parallel (21). These networks activate signal cascades and intermediates that include mitogen-activated protein kinases (MAPKs), PI3K, AKT, Smads, RhoB, c-Fos, and RAS (93), which then regulate EMT-TFs. RTKs are common initiation sites for signaling that induces EMT-TF activity.

### AXL

AXL is an archetypal RTK associated with EMT (94–96) and with worse outcomes in multiple tumor types (71, 94, 97, 98). Consistent with poor outcomes, AXL expression also is associated with metastasis and resistance to therapy (54, 96). AXL is a member of the TAM (Tyro3, AXL, MerTK) family of RTKs (99). Its ligand, growth arrest-specific gene 6 (GAS6) induces AXL signaling by stimulating the auto-phosphorylation of several tyrosine residues of AXL, which function as docking sites for multiple substrates including PI3K, phospholipase C, and c-SRC (100, 101). Additionally, AXL can be activated by forming heterodimers with non-TAM family proteins, such as EGFR, PDGFR, or another TAM family member (71). Elevated AXL expression is found in multiple cancer types, including lung, breast, ovarian, gastric, colon, pancreatic, and prostate (71–73, 94, 95, 97, 102, 103). AXL expression is induced by drivers of EMT, for example TGFβ, and is generally associated with markers of EMT including N-cadherin and vimentin (104, 105).

Our lab and others have shown that AXL expression in RAS-driven cancers, such as PDAC, maintains epithelial plasticity (96). GAS6-AXL signal transduction is required to maintain epithelial-mesenchymal plasticity traits of PDAC (96). When AXL was inhibited in GEMMs of pancreatic cancer, Ludwig et al. (54) observed an increase of epithelial differentiated tumor cells. In addition to chemotherapy resistance, AXL has been strongly implicated in resistance to targeted therapy such as EGFR and PI3K/AKT inhibitors (72, 73).

### Oncogenic KRAS

*RAS* genes (*HRAS, KRAS*, and *NRAS*) are the most frequently mutated gene family in cancer (106). Of these, *KRAS* is the most mutated (86% of all *RAS*-mutant cancers), followed by *NRAS* (12%), and *HRAS* (4%) (107). *KRAS* mutations are frequent in PDAC, lung, and colorectal cancers, and also occur in other cancers such as multiple myeloma (2, 108).

**Figure 2:**
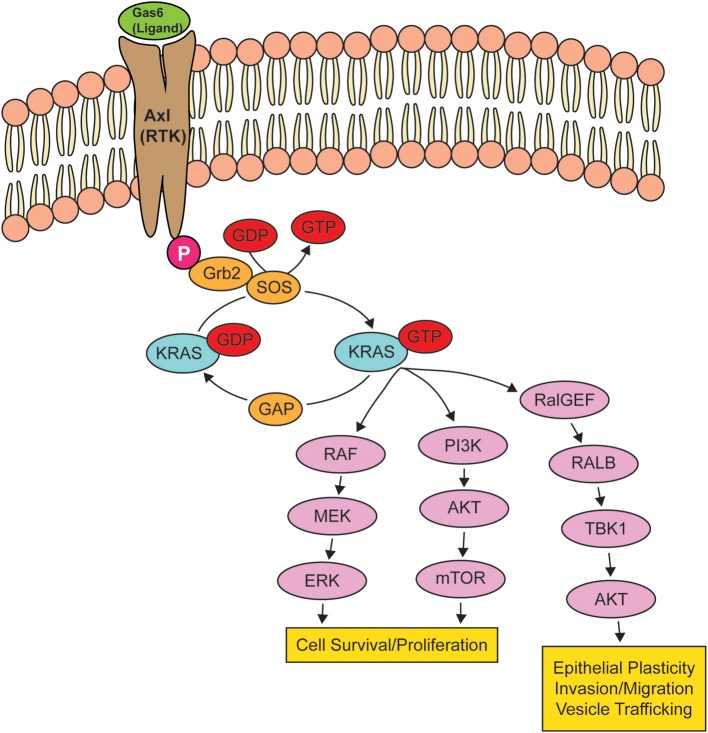
Oncogenic KRAS effector pathways. When a receptor tyrosine kinase (RTK) is activated by its ligand, KRAS binds to GTP, rendering it active until the GTP hydrolyzes to GDP, turning KRAS off. When *KRAS* is mutated, KRAS remains bound to GTP, leading to the overstimulation of KRAS signaling pathways, resulting in cell survival and proliferation, epithelial plasticity, and migration. The activation of RTK AXL by GAS6 is shown as a potential signaling pathway that can drive an epithelial-to-mesenchymal transition via the activation of KRAS.

KRAS, a small GTPase, functions as a molecular switch, cycling between an active guanosine triphosphate (GTP)-bound and inactive guanosine diphosphate (GDP)-bound states (109). In non-transformed cells, RAS is typically GDP-bound and inactive, but upon activation of RTKs, there is a rapid activation of RAS-GTP, leading to the activation of intracellular signaling networks that promote growth, proliferation, and migration (110) (Figure 2). Because KRAS-activating mutations cluster around the nucleotide-binding pocket (2), these mutations cause RAS to be persistently GTP-bound and constitutively active, resulting in the hyperactivation of signaling networks to drive cancer growth and progression (111).

Multiple RTKs, including AXL and EGFR, can activate KRAS (112). Signaling networks downstream of RAS such as ERK/MAPK and PI3K/AKT can mediate mutant *Ras*-induced EMT, such that the inhibition of MEK1 or AKT (113) can reverse RAS-stimulated epithelial plasticity. Genovese et al. (114) completed a gene set enrichment analysis of highly metastatic and poorly metastatic clonal cells lines isolated from a GEMM of PDAC, i.e., KPfC mice (*KRAS*^*LSLG*12*D*/+^*; Trp53*^*Lox*/*Lox*^*; Pdx1*^*Cre*/+^). Their analysis revealed that “metastasis-low” clones exhibited a downregulating of *KRAS* signature genes, whereas “metastasis-high” clones exhibited a higher expression of *KRAS* signature genes (114). After validation through *in vivo* lineage tracing, their study demonstrated that in PDAC, cells reside in a spectrum of epithelial-mesenchymal states where mesenchymal cells activate KRAS signaling at a higher level.

Other genome-sequencing studies revealed genetic heterogeneity beyond a few frequently mutated drivers in human PDAC (115–121). The heterogeneity in genomic changes makes it challenging to link definitive genomic alterations to biological, morphological, or clinical phenotypes (116, 121). Despite these challenges, Mueller et al. (37), found that the gene dosage of *KRAS G12D* in human and mouse PDAC correlated with a markedly increased metastatic potential and a mesenchymal phenotype. These results link the aggressive mesenchymal PDAC subtype with the highest dosage of mutant *KRAS* and *Ras*-related transcriptional programs. Additionally, oncogenic *Ras* is closely associated with resistance to drug therapy and pathways that drive PDAC initiation, progression, and metastasis.

### TBK1

Although the majority of RAS effector-targeted therapies inhibit the RAF and PI3K signaling networks, the RALGEF pathway encompassing RALA and RALB GTPases are more consistently activated than RAF or PI3K in human PDAC (122, 123). Additionally, it has been demonstrated in human cell lines that RALGTPase activation is essential for RAS-induced transformation in a spectrum of human epithelial cells and that RALGTPase activation alone is sufficient to induce a tumorigenic phenotype in some settings (124, 125). Given that RAS signaling is a driver of epithelial plasticity and that the RALGEF pathway is a critical effector of RAS, investigating RALGEF signaling has the potential to reveal novel targets involved in epithelial plasticity, metastasis, and therapy resistance in *RAS*-mutant tumors.

The serine/threonine protein kinase TANK-binding kinase 1 (TBK1) is an atypical Ikβ kinase, that together with its homolog, IKKε, contributes to innate immunity by activating interferon regulatory factor 3/7 (IRF3/7) thereby inducing type 1 interferon gene expression in response to pathogen exposure (126, 127). Additionally, TBK1 kinase activity supports cell growth, self-renewal, pathogen clearance, and organelle function (128–131). TBK1 is a constituent of the RAL pathway and is crucial to the induction and progression of RAS-driven cancers (105, 130, 132, 133). Additionally, TBK1 has been linked to the survival of mutant KRAS-expressing cells (128) and can directly activate AKT (130). The importance of RALB and TBK1 to RAS-induced lung cancer was confirmed in a RNA inhibitor screen of synthetic lethal partners of oncogenic KRAS, where RALB and TBK1 were identified as top targets (132). Further, Cooper et al. (134) screened 100 NSCLC lines for sensitivity to TBK1 inhibitors Bx795 and compound II to tease out biological features of TBK1-dependent cell lines. Sensitivity profiles correlated strongly with profiles of multiple inhibitors of the AKT/mTOR pathway, particularly in mutant *KRAS* NSCLC lines, suggesting a mechanistic interaction between TBK1 and the mTOR pathway (134). Further analysis of TBK1 inhibitor (TBK1i)-sensitive cell lines revealed mutations in RAS family members and increased mesenchymal gene expression compared to TBK1i-resistant cell lines, which had a more differentiated gene expression profile.

In support of the contribution of TBK1 to RAS-induced EMT, we reported that TBK1 expression is associated with a poor prognosis in pancreatic cancer patients (135). Furthermore, we found that the loss of TBK1 function resulted in reduced invasion, migration, and tumor growth, and reduced metastatic events in preclinical models of mutant *KRAS* PDAC, indicating that TBK1 actively contributes to pancreatic cancer progression (105). In fact, one of the most significant and top dysregulated gene networks distinguishing *TBK1* WT and *TBK1*-mutant tumors was the cancer/cellular movement networks, including many genes involved in EMT. In comparison with *TBK1* WT tumors, tumors from *TBK1* mutant mice showed a trend toward higher expression of epithelial markers and lower expression of mesenchymal markers; this trend was confirmed at the protein level (105). Mechanistic studies established that TBK1 promotes EMT downstream of AXL in PDAC, in a RAS-RALB dependent manner (105). Although the precise mechanism of how TBK1 promotes EMT is unclear, evidence suggests that TBK1 can directly activate AKT (130), which can drive EMT via the induction of EMT-TFs (e.g., Snail and Slug) (38, 136, 137). Further studies are needed to delineate the whether the interaction between TBK1 and AKT is critical to the mesenchymal phenotype of tumor cells in PDAC. The identification of additional TBK1 substrates that might promote EMT programs is also needed.

In contrast, knockdown of TBK1 in estrogen receptor α-positive (ERα) breast cancer cells resulted in enhanced tumorigenesis and lung metastasis in part by increasing EMT (138). Further studies are required to investigate if this pathway is dependent on oncogenic RAS. Another group observed that TBK1 is active in mutant *NRAS* melanoma and promoted migration and invasion of these cells (139), suggesting that RAS-driven epithelial plasticity may be active in the presence of other RAS isoform-driven cancers. Regardless, these studies suggest that therapies targeting TBK1 could be used to reduce EMT in *Ras*-mutant tumors.

### cGAS-STING and Innate Immunity in EMT

In agreement with the concept that TBK1 loss affects antitumor immunity, studies by the Cantley (140) and Barbie (133) groups have reported that immune evasion and metastatic behavior are associated with the cGAS/STING/TBK1 innate immune pathway in cancer cells (133, 140, 141). Canadas et al. (133) revealed that mesenchymal tumor subpopulations with high AXL expression and low histone-lysine N-methyltransferase levels trigger the expression of a specific set of interferon-stimulated antisense endogenous retroviruses (ERVs). These ERVs were present in human cancer cells that produced tumors with hyperactive innate immune signaling, myeloid cell infiltration, and utilized immune checkpoint pathways. Therapeutically, this may have important implications for immune oncology drug combinations. In the second study, Bakhoum et al. (140) found that chromosomal instability (CIN) of cancer cells, promoted cellular invasion and metastasis through the presence of double-stranded DNA in the cytosol. Clustering of tumor cells via EMT genes accurately classified most cells according to their CIN status and revealed that CIN-high cells expressed mesenchymal markers. This CIN-high population also exhibited increased migratory and invasive behavior *in vitro*, underwent actin cytoskeletal reorganization, and stained positive for mesenchymal markers such as vimentin and β-catenin. Additionally, cells derived from metastases more frequently exhibited cytoplasmic micronuclei than CIN-low or primary tumor-derived cells. These studies showed that cytosolic DNA activates the cGAS/STING pathway to mediate EMT, invasion, and metastasis (140). Under normal conditions, the cGAS-STING pathway functions as an innate cellular defense mechanism against viral infections. Once STING activates TBK1, TFs such as IRF3 and NF-κB are phosphorylated and translocate to the nucleus (142), where they mediate the transcription of inflammatory genes (143–146). In human breast and lung cancer-derived cell lines, chronic cGAS-STING activity resulting from chromosome instability has been shown to drive migration, invasion, and metastasis (140). Additionally, CIN can result in elevated mutant *KRAS* gene dosage in pancreatic cancer, which can drive higher expression of EMT genes and increase metastasis (37).

Similar to epithelial plasticity, CIN has been implicated in treatment resistance by generating heterogeneity within the tumor that enhances natural selection, thereby promoting tumor cell survival, immune evasion, drug resistance, and metastasis (37, 147–152). Given the widespread nature of CIN in human cancer, therapies targeting CIN and cGAS/STING have therapeutic potential to reduce therapy resistance and reduce metastasis.

### Downstream Transcriptional Networks of Epithelial Plasticity

EMT is thought to be regulated largely through changes in the expression of genes necessary for the epithelial state, such as adherens junctions and tight junction components, which are transcriptionally repressed through the activation of EMT TFs including Snail, Twist, and Zeb (153). As previously mentioned, EMT can be induced by many signaling factors, such as TGFβ, EGF, FGF, HGF, NOTCH, and Wnt ligands. These factors initiate signaling cascades, leading to the expression of one or more EMT-TFs, which inhibit E-cadherin transcription by binding to E-boxes within the E-cadherin promoter region (154, 155).

EMT-TFs are often associated with poor patient outcomes. In resected PDAC, nearly 80% of tumors expressed moderate to strong levels of SNAI1, while only 50% showed SNAI2 expression, and very few expressed TWIST (156). Additionally, ZEB1 expression in pathologic specimens correlated with advanced tumor grade and worse outcomes (157, 158). Functions for individual EMT-TFs in different cancers have been described: for ZEB1 and ZEB2 in melanoma (159, 160), Snail and Slug in breast cancer (161), and for Sox4 (162), and Prrx (163) in PDAC. These functions can be tissue-specific, as demonstrated by the different functions of Snail in the metastasis of breast cancer (164) and PDAC (55). Such functional diversity of EMT-TFs suggests that distinct EMT programs operate in different tissues during tumor progression. With this in mind, therapeutic strategies targeting EMT-TFs should consider tissue context and target multiple factors simultaneously (112).

ZEB1 is a zinc finger/homeodomain protein that is associated with EMT and tumor progression. ZEB1 functions as a transcriptional activator by binding to CtBP co-repressors, histone acetyl-transferase TIP60, chromatin remodeling ATPase BRG1, and SIRT1, a histone deacetylase (21). Larsen et al. (165) found that ZEB1-induced EMT was crucial for the development of NSCLC but required premalignant oncogenic mutations such those for *KRAS*. Moreover, they found that ZEB1-driven EMT was a crucial early event in the progression of human bronchial epithelial cells to malignancy (165). These results supported previous *in vitro* (166) and *in vivo* (167–170) studies that established ZEB1 as a driver of EMT in lung cancer tumorigenesis. In PDAC, Krebs et al. (112) demonstrated that ZEB1 is a key driver of PDAC progression from early tumorigenesis to late-stage metastasis, highlighting the important contribution of EMT activation in these processes (112).

Beyond the levels of mRNAs, EMT-TFs can alter chromatin to achieve the stable, long-term silencing of epithelial genes required for complete EMT (171). Snail, an EMT-TF, can recruit a series of chromatin-modifying enzymes to the E-cadherin promotor to erase a mark of active transport and replace it with a trimethylated H3K9 mark that promotes the recruitment of DNA methyltransferases, causing CpG methylation of the promoter and formation of a constitutive heterochromatin resistant to transcription activation (172). Additionally, TFs of the Zeb family form a double-negative feedback loop with the miR-200 family of microRNAs (miRNA), causing this regulatory loop to operate as a switch between epithelial and mesenchymal states in a variety of tumor types (173–175). Similarly, Snail represses the expression of miR-34, a miRNA that binds to the 3′ UTR of Snail mRNA to mark it for degradation (176).

## Targeting KRAS Signaling as a Therapeutic Approach

### Direct Targeting of KRAS

Targeting RAS proteins was first attempted when the proteins were shown to be modified and rendered functional by farnesylation (177–179). This initiated the launch of identifying compounds that block farnesyl transferase activity. Farnesyl transferase inhibitors were developed with impressive potency and selectivity, but they failed to show efficacy in the clinic (180). Another approach that has been considered is the development of a GTP antagonist. However, due to the picomolar affinity of GTP and RAS and the millimolar concentration of GTP in the cell, GTP antagonists had long been deemed impossible (111) until recently. In 2013, the dream of directly targeting RAS was re-imagined when Shokat and colleagues identified compounds that bind covalently and specifically to *KRAS G12C* (181). Lead compounds were further developed by Wellspring Biosciences, who showed that the compounds ARS853 and ARS1620 inhibit *KRAS G12C* effectively and specifically in cells and animals (182, 183). The first *KRAS G12C* inhibitor to enter clinical trials is Amgen 510 (Table 1). Multiple groups are working to create improved G12C-targeted compounds with better RAS-GTP destabilizing activity (184, 185). These studies have reinvigorated the field and initiated research efforts, such as the NCI-supported RAS initiative.

**Table 1:** Clinical trials targeting KRAS, AXL, and TBK1.

**Target**	**Drug**	**Disease**	**Trial phase**	**Results**	**Identifier**
KRAS G12C	AMG 510	NSCLC	1/2	Ongoing	NCT03600883
KRAS G12C	MRTX849	Advanced solid tumors	1/2	Ongoing	NCT03785249
AXL	Bemcentinib (BGB324)	Glioblastoma	1	Ongoing	NCT03965494
AXL	Bemcentinib (BGB324)	Pancreas	1/2	Ongoing	NCT03649321
AXL	Bemcentinib (BGB324)	NSCLC	2	Ongoing	NCT03184571
AXL	Bemcentinib (BGB324)	NSCLC	1/2	Status unknown	NCT02424617
AXL	Bemcentinib (BGB324)	Malignant mesothelioma	2	Ongoing	NCT03654833
AXL	Bemcentinib (BGB324)	NSCLC	1	Ongoing	NCT02922777
AXL	Bemcentinib (BGB324)	TNBC	2	Completed	NCT03184558
AXL	Bemcentinib (BGB324)	Melanoma	1/2	Ongoing	NCT02872259
AXL	Bemcentinib (BGB324)	Acute myeloid leukemia	2	Ongoing	NCT03824080
AXL	TP-0903	NSCLC, colorectal, ovarian, melanoma	1	Ongoing	NCT02729298
AXL	TP-0903	Leukemia, lymphoma	1/2	Ongoing	NCT03572634
TBK1	Amlexanox	Type 2 diabetes	2	Finished recruitment	NCT01842282
TBK1	Amlexanox	Type 2 diabetes	2	Optimal drug dose wasn't reached.	NCT01975935

*NSCLC, non-small cell lung cancer; TNBC, triple-negative breast cancer*.

Although this recent breakthrough suggests that targeting *KRAS G12C* may be effective, it is possible that this targetable allele may be an outlier (186). *KRAS G12C* is rarely mutated in KRAS-addicted cancers and it is likely that *KRAS G12D* and *G12V*, the most common mutant *KRAS* alleles, will be more challenging to specifically inhibit (187). As a result, the development of therapeutic strategies that either inhibit RAS effector signaling elements, such as TBK1, or inhibit elements that can activate *RAS*, such as AXL, remain an attractive therapeutic alternative.

### Targeting AXL and TBK1 as a Therapeutic Strategy for KRAS-Driven Cancers

Due to its implication in metastasis, EMT, and drug therapy resistance, large efforts are focused on pharmacologically inhibiting AXL. In fact, multiple strategies are being tested clinically, including blocking GAS6 or AXL with monoclonal antibodies and small molecules (99, 188). One of the most advanced selective AXL inhibitors to date is bemcentinib (BGB324), developed by BerGenBio ASA. BGB324 has been investigated by our group in preclinical models of late-stage PDAC and shown promising therapeutic effects in enhancing gemcitabine efficacy and reducing metastasis (54). Other groups have also investigated BGB324, where it has been found to have antitumor, antimetastatic, and therapy-sensitizing effects in preclinical models of pancreatic cancer, breast cancer, glioblastoma, prostate cancer, chronic myeloid leukemia, ovarian cancer, and uterine serous cancer (189–195). Recently phase II clinical trials have begun to enroll patients using bemcentinib in multiple cancer types as a single agent or in combination with targeted or chemo- and immunotherapies (Table 1). Another selective AXL inhibitor is TP-0903, developed by Tolero Pharmaceuticals. In preclinical models, TP-0903 has been shown to have antitumor and therapy-sensitizing effects on multiple cancers, including neuroblastoma, leukemia, and lung cancer (196–199). TP-0903 is currently being evaluated clinically in multiple indications (Table 1).

For TBK1 to be a relevant target in the clinic, it will be necessary to evaluate the therapeutic efficacy of TBK1 inhibition in preclinical cancer models. Currently there are at least six distinct small molecules that inhibit TBK1, including BX795, compound II, CYT387, MRT67307, GSK2292978A, and Amlexanox, although none are highly selective. Currently, Amlexanox is the only TBK1i known to enter clinical testing, which is in a phase 2 study for the treatment of type 2 diabetes, non-alcoholic fatty liver disease, or obesity (Table 1). Further investigations and better inhibitors will be needed before TBK1 can be directly targeted in RAS-driven cancer in preclinical and clinical settings. Moving forward, it will be vital to understand the distinct function of TBK1 in each relevant cell type within tumors. As mesenchymal tumor cells express high levels of active TBK1 (105) and are associated with aggressive disease, metastasis, and poor patient outcomes (30), targeting TBK1 in RAS-driven cancers is a promising alternative strategy to reduce the tumor-promoting effects of KRAS-driven EMT.

## Conclusions and Future Perspectives

EMT is a key cellular program that is activated by KRAS and thus contributes to tumor progression by enhancing tumor cell survival, tumor cell dissemination, and therapy resistance and has a strong association with worse clinical prognosis in many KRAS-driven cancers. Because KRAS is not currently an amenable target for many of these KRAS-driven cancers, targeting KRAS effector signaling is an attractive alternative. With this in mind, pharmacologically targeting the pathways that contribute to KRAS-driven EMT is worth considering as a strategy to improve response to standard therapy and reduce clinical progression, therapy resistance, and metastasis.

Despite significant evidence that EMT directly contributes to tumor progression, several studies have suggested EMT is not required for the metastatic spread of PDAC and breast cancer (55, 57, 200, 201). For example, most metastatic lesions are known to exhibit epithelial features, an observation that seems to be at odds with EMT as a prerequisite for metastasis (30, 202, 203). As such, the importance of EMT in cancer biology has long been questioned (204).

Epithelial plasticity not only includes the process of EMT, but also the reverse, mesenchymal-to-epithelial transition or MET. Recent evidence suggests that MET is required for successful metastatic colonization, although it remains unknown whether the tissue-specific adaptations are acquired thorough epigenetic or genetic means. Distant metastases in carcinoma patients often present with epithelial features having a similar histology as the tissue of origin (205, 206). These observations support that epithelial plasticity lies at the heart of tumor development and progression, and that such plasticity is necessary for tumor cell survival and colonization. It has become increasingly evident that EMT encompasses a range of hybrid plastic states, a phenotype coined as “partial EMT” (36, 207, 208). Because partial EMT is not well-defined, it is unclear whether this hybrid status signifies a transitional phase during EMT or represents its own state. Similarly, using a mouse model of PDAC, the Stanger group has shown that individual tumors can activate different plasticity programs, such as “classical EMT” which involves transcriptional repression and an alternative program in which the epithelial state is lost post-transcriptionally (36). These plasticity programs were associated with either single-cell invasion or collective invasion, respectively (36). It is unclear what underlies this phenotypic heterogeneity, considering the tumors investigated in this study had the same oncogenic drivers (TP53 and KRAS). Perhaps the only difference between the states is the tumor microenvironment, as Aiello et al. found that when partial EMT cells are exposed to TGFβ, they execute a classic EMT program (36, 209). This constant plastic state may partially explain the intratumoral heterogeneity that is often seen in carcinomas such as PDAC (210–212).

The chronic activation of an EMT program within a tumor may depend on paracrine signals within the tumor microenvironment, dictating whether the tumor cells undergo EMT or MET. Because these cells exist in a plastic state, it is possible that these tumor cells readily revert their phenotype based on a microenvironment-specific context and factors (36, 205, 213, 214). One challenge impeding current *in vivo* studies is the difficulty of distinguishing carcinoma cells that have undergone EMT from fibroblasts or other mesenchymal cells that are normally found in the tumor stroma. To combat this, many labs have begun to use single-cell sequencing technology in KRAS-driven cancers such as PDAC to investigate EMT *in vivo* (215). Additionally, current *in vivo* lineage-tracing technology has not settled the debate between the importance of collective migration and/or EMT for metastatic dissemination. Additionally, the mechanisms of invasion and metastatic potential and their correlation with clinical outcome has yet to be defined. Regardless, epithelial plasticity remains as an indispensable feature in multiple phases of human cancer in an oncogene- and tissue-specific manner.

## Author Contributions

EA and WD wrote the manuscript. RB reviewed and revised the manuscript.

### Conflict of Interest

RB receives research support from BerGenBio ASA and Tolero, companies developing Axl inhibitors. The remaining authors declare that the research was conducted in the absence of any commercial or financial relationships that could be construed as a potential conflict of interest.
